# Racial and Ethnic Differences in Insurance Outcomes After Job Loss During the First Year of the COVID-19 Pandemic

**DOI:** 10.1001/jamahealthforum.2023.0168

**Published:** 2023-03-31

**Authors:** Zhanji Zhang, José J. Escarce, Dennis Rünger, James Campbell, Peter J. Huckfeldt

**Affiliations:** 1 Department of Applied Economics, University of Minnesota, Minneapolis, Minnesota; 2 Division of General Internal Medicine and Health Services Research, Department of Medicine, David Geffen School of Medicine at UCLA, Los Angeles, California; 3 Department of Health Policy and Management, Fielding School of Public Health, University of California, Los Angeles; 4 Division of Health Policy and Management, School of Public Health, University of Minnesota, Minneapolis, Minnesota

## Abstract

This cohort study uses national survey data to assess the racial and ethnic differences in insurance coverage after job loss during the first year of the COVID-19 pandemic.

## Introduction

Due to the COVID-19 pandemic, unemployment soared from 3.5% in February 2020 to 14.7% in April before falling to 6.7% in December.^1^ Research found that uninsurance rose among working-age adults due to falling employer-sponsored insurance (ESI) among newly unemployed workers that was only partially offset by Medicaid.^2,3^ We assessed racial and ethnic differences in uninsurance and coverage sources among working-age adults who worked in 2019 and 2020 (continuously employed) and who worked in 2019 but were unemployed in 2020 (newly unemployed).

## Methods

In this cohort study, we used 2019-2020 longitudinal data from the National Health Interview Survey (NHIS) (publicly available data not requiring informed consent). The 2019 interviews occurred throughout the year, whereas 2020 interviews occurred between August and December. The study was approved by the University of California, Los Angeles, institutional review board and followed the STROBE reporting guideline.

We used linear probability models to analyze 3 insurance outcomes: uninsurance, Medicaid, and private. We stratified analyses by participant-reported race and ethnicity (using the NHIS recode variable) of all working-age adults (18-64 years) and those with a job in 2019, comparing continuously employed and newly unemployed (eMethods in Supplement 1). Independent variables included year, person fixed effects, and interaction of employment status and year. We estimated models using survey commands accounting for weighting, stratification, and clustering at the primary sampling unit level. Statistical analyses were performed between June 2022 and January 2023, using Stata, version 17.0 software (StataCorp LLC).

## Results

The Table presents demographic details and insurance coverage for the sample. Unweighted and weighted sample sizes were 4613 and 126 202 767 for continuously employed and 545 and 16 575 007 for newly unemployed participants. Compared with continuously unemployed participants, those newly unemployed were more likely to be women (61.4% vs 38.6% men) and less likely to be White (8.3% Asian, 16.6% Black, 21.9% Hispanic, 4.7% other vs 48.5% White), had lower educational attainment and income, and were less likely to be privately insured in 2019 and more likely to have Medicaid.

**Table.** Sociodemographic Characteristics and Insurance and US Citizenship Status of Study Participants, 2019-2020^a^

Characteristic	Participants, % (95% CI)			
	All working-age adults	Working-age adults who worked in 2019	Continuously employed	Newly unemployed
Age, mean (SD), y	40.2 (13.3)	39.9 (12.7)	39.9 (12.4)	40.0 (14.5)
Sex				
Female	50.8 (49.1-52.5)	47.4 (45.5-49.3)	45.7 (43.7-47.8)	61.4 (56.2-66.3)
Male	49.2 (47.5-50.9)	52.6 (50.7-54.5)	54.3 (52.2-56.3)	38.6 (33.7-43.8)
Race and ethnicity				
Asian	6.1 (5.3-7.0)	6.0 (5.1-7.0)	5.8 (4.9-6.7)	8.3 (5.2-13.0)
Black	12.6 (11.1-14.2)	11.0 (9.6-12.6)	9.7 (8.4-11.3)	16.6 (12.4-21.9)
Hispanic	18.8 (16.8-21.0)	18.9 (16.8-21.2)	18.4 (16.3-20.8)	21.9 (17.5-27.1)
White	59.7 (57.2-62.1)	61.2 (58.7-63.7)	63.5 (61.0-66.0)	48.5 (43.0-54.1)
Other^b^	2.9 (2.0-4.0)	2.9 (2.2-3.8)	2.6 (1.9-3.4)	4.7 (2.8-7.9)
Education, %				
High school or less	38.5 (36.6-40.4)	34.1 (32.1-36.1)	32.4 (30.4-34.6)	44.2 (38.6-49.8)
Some college	32.0 (30.5-33.6)	32.4 (30.6-34.2)	32.3 (30.4-34.2)	35.1 (30.2-40.4)
College degree or more	29.5 (27.9-31.1)	33.5 (31.6-35.4)	35.3 (33.4-37.3)	20.7 (17.0-24.9)
Income, % FPL				
≤138	18.8 (17.5-20.3)	12.5 (11.1-14.0)	10.8 (9.5-12.3)	22.6 (18.3-27.7)
139-199	10.5 (9.5-11.5)	9.9 (8.9-11.1)	9.2 (8.1-10.5)	13.8 (10.1-18.5)
200-399	30.9 (29.4-32.5)	32.3 (30.5-34.1)	32.3 (30.4-34.3)	32.8 (27.9-38.1)
≥400	39.8 (38.0-41.5)	45.3 (43.4-47.2)	47.7 (45.6-49.8)	30.8 (25.9-36.1)
Insurance				
2019				
Uninsured	14.4 (13.0-15.8)	13.9 (12.5-15.5)	13.6 (12.1-15.3)	14.5 (10.7-19.3)
Medicaid	13.2 (11.9-14.7)	7.8 (6.6-9.1)	6.4 (5.3-7.7)	18.3 (14.2-23.2)
Private	63.0 (61.2-64.8)	70.9 (69.0-72.8)	73.8 (71.7-75.7)	50.8 (45.2-56.4)
Exchange	4.8 (4.1-5.6)	4.8 (4.0-5.8)	4.3 (3.5-5.2)	9.6 (6.5-13.9)
Other	4.6 (4.0-5.3)	2.6 (2.1-3.1)	2.0 (1.6-2.5)	6.9 (4.9-9.5)
2020				
Uninsured	13.9 (12.6-15.3)	13.6 (12.3-15.1)	12.5 (11.1-14.0)	20.9 (16.3-26.3)
Medicaid	13.1 (11.8-14.5)	8.5 (7.4-9.8)	6.3 (5.3-7.5)	23.5 (18.7-29.1)
Private	62.4 (60.6-64.2)	69.8 (67.9-71.7)	74.1 (72.3-75.9)	39.1 (34.0-44.5)
Exchange	4.9 (4.2-5.7)	4.9 (4.1-5.9)	4.4 (3.7-5.3)	9.4 (6.2-13.9)
Other	5.8 (5.0-6.6)	3.1 (2.6-3.8)	2.6 (2.2-3.2)	7.2 (4.6-10.9)
Citizenship				
2019				
Non-US	11.4 (10.1-12.8)	10.8 (9.5-12.2)	10.2 (8.8-11.7)	13.7 (10.1-18.3)
US	88.6 (87.2-89.9)	89.2 (87.8-90.5)	89.8 (88.3-91.2)	86.3 (81.7-89.9)
2020				
Non-US	11.4 (10.1-12.8)	10.8 (9.5-12.2)	10.2 (8.8-11.7)	13.7 (10.1-18.3)
US	88.6 (87.2-89.9)	89.2 (87.8-90.5)	89.8 (88.3-91.2)	86.3 (81.7-89.9)
Unweighted No. of participants	6717	5323	4613	545
Weighted No. of participants	192 094 382	147 871 245	126 202 767	16 575 007

Abbreviation: FPL, federal poverty level.

^a^ Sociodemographic characteristics are from 2019. Estimates incorporated National Health Interview Survey sample weights and survey design parameters. Working age sample includes individuals aged 18 to 64 years. A small number of respondents who worked in 2019 but did not report their job status in 2020 were included in the analysis of working-age adults who worked in 2019 but not in the analyses of continuously employed and newly unemployed participants.

^b^ The “other” category included non-Hispanic American Indian or Alaska Native, with or without any other group; and other single and multiple races. A detailed explanation of how race and ethnicity were categorized is provided in the eMethods in Supplement 1.

Uninsurance and coverage sources were unchanged from 2019 to 2020 for all working-age adults, those employed in 2019, and those continuously employed, regardless of race and ethnicity (Figure). For newly unemployed participants, private insurance declined in 2019-2020 (−11.7 [95% CI, −16.9 to −6.5] percentage points), which was only partially offset by a rise in Medicaid coverage (5.2 [95% CI, 1.1-9.3] percentage points), resulting in higher uninsurance (6.4 [95% CI, 1.7-11.1] percentage points). Findings differed by race and ethnicity: although the decline in private insurance was offset by higher Medicaid coverage among newly unemployed White participants, there was no offsetting increase in Medicaid among newly unemployed Black or Hispanic participants (even when we excluded non–US-born Hispanic workers living in the US for <5 years), resulting in growth in uninsurance for both groups (Figure).

**Figure.**
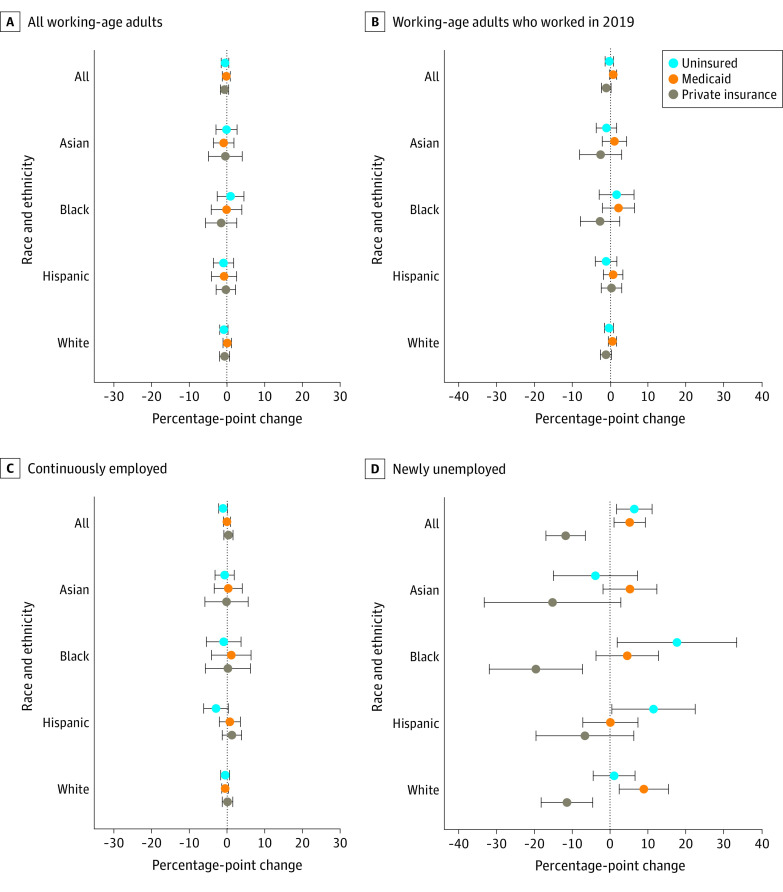
Changes in Health Insurance Status From 2019 to 2020

## Discussion

Whereas the decline in ESI in 2020 was offset by an increase in Medicaid coverage among newly unemployed White working-age adults, there was no such rise among newly unemployed Black and Hispanic workers. Black (46%) and Hispanic (36%) individuals were more likely than White individuals (33%) to have lived in states without Medicaid expansion by 2020, which could contribute to their higher uninsurance,^4^ but the differences are probably too small, especially for Hispanic individuals, to fully explain our findings. Further, Medicaid enrollment growth in 2020 was similar in expansion and nonexpansion states.^5^ Many non–US-born Hispanic individuals face a 5-year wait before becoming eligible for Medicaid, but our results were unchanged after excluding Hispanic individuals living in the US less than 5 years. Limitations include lack of precision in our estimates due to small sample sizes and the coarseness of our unemployment measure. The most likely explanation for our findings is that, during the pandemic’s first year, Black and Hispanic individuals who lost ESI were more adversely impacted than their White peers by structural and administrative barriers to Medicaid enrollment.^6^

## Notes

Supplement 1. **eMethods.** Technical Appendix

Supplement 2.
Data Sharing Statement
