# Supplementary material for: Racial and Ethnic Differences in Insurance Outcomes After Job Loss During the First Year of the COVID-19 Pandemic
Source: JAMA Health Forum. 2023 Mar 31;4(3):e230168. doi: 10.1001/jamahealthforum.2023.0168 (PMC10066457; doi:10.1001/jamahealthforum.2023.0168)
Supplement: Supplement 1. — eMethods. Technical Appendix [file jamahealthforum-e230168-s001.pdf]

Supplemental Online Content

Zhang Z, Escarce JJ, Rünger D, Campbell J, Huckfeldt PJ. Racial and ethnic differences in insurance outcomes after job loss during the first year of the COVID-19 pandemic. *JAMA Health Forum*. 2023;4(3):e230168. doi:10.1001/jamahealthforum.2023.0168

eMethods. Technical Appendix

This supplemental material has been provided by the authors to give readers additional information about their work.

© 2023 Zhang Z et al. *JAMA Health Forum.*

eMethods. Technical Appendix

1. **Measurement of race and ethnicity**

We categorized race and ethnicity using the NHIS recode variable[1], "HISPALLP_A". HISPALL_A categorizes participants as: Hispanic, Non-Hispanic (NH) White only, NH Black/African American only, NH Asian only, NH American Indian Alaska Native (AIAN) only, NH AIAN and any other group, and "Other single and multiple races". We directly used HISPALLP_A categories for Hispanic, NH White only, NH Black/African American only, and NH Asian only. We combined NH AIAN only, NH AIAN and any other group, and "Other single and multiple races" into a single "Other" category due to the small sample sizes in those groups. For brevity, we labeled these five categories in the research letter as: Asian, Black, Hispanic, White, and Other.

2. **Measurement of job loss**

An important part of our analysis was identifying working age adults in the NHIS 2019 survey who were either continuously employed in 2019 and 2020 or employed in 2019 and newly unemployed in 2020. We identified working age adults based on individuals aged 18 to 64 in both 2019 and 2020 (using the "SRVY_YR" variable for survey year) using the NHIS variable "AGEP_A". We defined "continuously employed" individuals as those answering "Yes" to working in the past week in both the 2019 and 2020 NHIS, using the NHIS recode variable "EMPWRKLSWK_A" (which takes on the values of Yes, No, or Don't Know). We identified "newly unemployed" individuals as individuals answering "Yes" to working in the past week in 2019 but answering "No" in 2020, again using EMPWRKLSWK_A.

---

[1] Recode indicates that the NHIS constructed the variable based on survey responses, but the variable does not reflect the raw, untransformed survey responses.

© 2023 Zhang Z et al. *JAMA Health Forum.*

3. Measurement of insurance coverage

The binary insurance outcomes in our analysis were "uninsurance" and enrollment in different types of health insurance. We created hierarchical and mutually exclusive insurance coverage categories, in the following order: Medicaid, Exchange, Private Insurance, Uninsured, and Other. For example, if someone reported holding both Medicaid and private insurance, then we would code them has holding Medicaid coverage, as it is the highest level in the hierarchy, but not private insurance.

The outcome "Medicaid enrollment" was based on the NHIS recode variable "MEDICAID_A" taking on the values of "Yes" (with or without information). The outcome "Health insurance exchange enrollment" was defined based on the NHIS recode variable "EXCHANGE_A" indicating that a respondent did have exchange coverage, excluding individuals with Medicaid coverage. Private insurance was based on the NHIS recode variable PRIVATE_A taking values of "Yes" (with and without information), excluding individuals reporting Medicaid or Exchange coverage. We found the percentage of individuals with private insurance reporting that their coverage was paid for by their employer or union was 73% in 2019 and 75% in 2020 (using the NHIS variables pln1pay2_a and pln2pay2_a, both taking values of "Mentioned"). "Uninsured" was defined based on the NHIS recode variable "COVER_A" indicating uninsured. "Other insurance" was a residual category including individuals without Medicaid, Exchange coverage, Private insurance, but who did have other coverage. This category included miliary-related coverage, Indian Health Service coverage, state-sponsored health plan, or other government programs.

© 2023 Zhang Z et al. *JAMA Health Forum.*

4. **Regression specifications**

For estimating changes in insurance coverage for all working age adults or all working age adults who worked in 2019 (i.e., Panels A and B of Figure 1), we estimated models such as equation 1, where *Insurance* was a binary indicator for either uninsurance, Medicaid, or private insurance for person *i* in year *t*, α is a constant, μ_i were person fixed effects, $Year2020_t$ was a binary variable equaling 1 if the year was 2020 and 0 if the year was 2019, and u_{it} was the error term.

$$Insurance_{it} = \alpha + \mu_i + \beta\, Year2020_t + u_{it} \qquad (1)$$

Panel A of Figure 1 displays coefficient estimates and 95% confidence intervals for β from estimating equation 1, indicating the average change in insurance from 2019 to 2020. We used the Stata "svy: reg" command that accounted for the sample weights (using the NHIS-provided longitudinal weight), the strata (the variable "PSTRAT"), and the primary sampling unit (the variable "PPSU"). Reflecting that all of our analysis samples were subsamples of the full NHIS sample, we used the Stata svy, subpop command to retain the full sample weighting information. We estimated equation 1 separately for each insurance outcome and by race and ethnicity.

For estimating changes in insurance coverage for continuously employed or newly unemployed participants, we estimated models such as equation 2, which were similar to equation 1 but added an interaction term between Year2020 and a binary variable "NewlyUnemp" which is a person-level binary indicator for someone who was employed in 2019 but newly unemployed in 2020.

$$Insurance_{it} = \alpha + \mu_i + \beta\, Year2020_t + \theta\, (Year2020_t \times NewlyUnemp_i) + u_{it} \qquad (2)$$

For the Panel C estimates (for the continuously employed), we estimated the model shown in equation (2) and reported the coefficient estimate and confidence interval for β indicating the

© 2023 Zhang Z et al. *JAMA Health Forum.*

change in 2020 for individuals who did not become unemployed. For the Panel D estimates (the newly unemployed), we calculated the sum of β and θ and estimated the 95% confidence interval yielding the overall change in insurance for individuals who became unemployed in 2020.

© 2023 Zhang Z et al. *JAMA Health Forum.*
