# Supplementary material for: Racial and Ethnic Differences in Insurance Outcomes After Job Loss During the First Year of the COVID-19 Pandemic
Source: JAMA Health Forum. 2023 Mar 31;4(3):e230168. doi: 10.1001/jamahealthforum.2023.0168 (PMC10066457; doi:10.1001/jamahealthforum.2023.0168)
Supplement: Supplement 2. — Data Sharing Statement [file jamahealthforum-e230168-s002.pdf]

Data Sharing Statement

Zhang. Racial and Ethnic Differences in Insurance Outcomes After Job Loss During the First Year of the COVID-19 Pandemic. *JAMA Health Forum*. Published March 31, 2023. doi:10.1001/jamahealthforum.2023.0168

Data
Data available: No
